# Associations of IL13 gene polymorphisms and immune factors with *Schistosoma haematobium* infection in schoolchildren in four schistosomiasis-endemic communities in Ghana

**DOI:** 10.1371/journal.pntd.0009455

**Published:** 2021-06-29

**Authors:** Margaret Sarpong-Baidoo, Michael F. Ofori, Elias Kwesi Asuming-Brempong, Eric Kyei-Baafour, Bright K. Idun, Isaac Owusu-Frimpong, Nana A. Amonoo, Queenstar D. Quarshie, Edward J. Tettevi, Mike Y. Osei-Atweneboana

**Affiliations:** 1 Biomedical and Public Health Research Unit, CSIR- Water Research Institute, Council for Scientific and Industrial Research, Accra, Ghana; 2 Department of Animal Biology and Conservation Sciences, College of Basic and Applied Sciences, University of Ghana, Legon, Accra, Ghana; 3 Department of Biomedical Sciences, College of Health and Allied Sciences, University of Cape Coast, Cape Coast, Ghana; 4 Department of Immunology, Noguchi Memorial Institute for Medical Research, College of Health Sciences, University of Ghana, Legon, Accra, Ghana; Weill Cornell Medical College, UNITED STATES

## Abstract

**Background:**

Schistosomiasis remains a major public health issue with over 90% of the prevalence rates recorded in Sub-Saharan Africa. In this study, the relationships between different interleukin gene polymorphisms (IL-13-591A/G, IL-13-1055C/T, IL-13-1258A/G) and *Schistosoma haematobium* infection levels were evaluated; as well as the host plasma antibodies and cytokine profiles associated with schistosomiasis infection.

**Methodology:**

A total of 469 school children aged 6 to 19 years from four schistosomiasis-endemic communities in Ghana were involved. Single urine and stool samples were obtained from each pupil, processed via sedimentation and Kato-Katz, and examined via microscopy for *Schistosoma* and soil-transmitted helminth (STH) eggs. Next, venous blood samples were drawn from 350 healthy pupils, and used to measure antibody and plasma cytokine levels by ELISA. Single nucleotide polymorphisms in the IL-13 gene were genotyped on 71 selected blood samples using the Mass Array technique.

**Principal findings and conclusion:**

The overall prevalence of urinary schistosomiasis was 21.11%. Community-level prevalences were 17.12%, 32.11%, 20.80%, and 15.32% for Asempaneye, Barikumah, Eyan Akotoguah, and Apewosika respectively. Generally, higher *S*. *haematobium* infection prevalence and intensity were recorded for participants with genotypes bearing the IL13-1055C allele, the IL13-591A, and the IL13-1258A alleles. Also, higher *S*. *haematobium* infection prevalence was observed among participants in the 12-14-year age group with the IL13-1055C, IL13-591A, and IL13-1258A alleles. Interestingly, higher STH prevalence was also observed among participants with the IL13-1055C, IL13-591A, and IL13-1258A alleles. Furthermore, the age-associated trends of measured antibodies and cytokines of *S*. *haematobium*-infected school-children depicted a more pro-inflammatory immune profile for pupils aged up to 1l years, and an increasingly anti-inflammatory profile for pupils aged 12 years and above. This work provides insight into the influence of IL-13 gene polymorphisms on *S*. *haematobium*, and STH infections, in school-aged children (SAC).

## Author summary

Schistosomiasis is a neglected tropical disease with an estimated morbidity of 2.54 million disability-adjusted life years (DALYS), ranking it third among the parasitic diseases after Malaria and Dengue fever (WHO, 2018). About a decade ago, the World Health Organization (WHO) and its partners committed to intensifying efforts at increasing coverage during annual mass chemotherapy programmes in affected countries. This has influenced the reduction of the estimated disease-associated morbidity from 4.5 million DALYs in 2002 (WHO, 2002) to the current rate. Despite these gains, there is still growing concern that the persistent and extensive use of Praziquantel (PZQ) (the only approved chemotherapeutic agent) in these campaigns may lead to the development of poorly-responding phenotypes of *Schistosoma sp*. Strategies therefore to mitigate such possibilities may prove very advantageous, while research efforts towards the development of an effective vaccine continues. There has been growing evidence in recent years that infection of inhabitants in endemic areas may be influenced not only by environmental, but also by genetic factors as well. Of particular interest in this regard, is the region in the human genome, 5q31-q33, which bears several genes associated with immune function. One such group within this region is the T-helper 2 (Th2) cluster of genes, which are responsible for the production interleukin-4 (IL-4), IL-5, and IL-13 during immune response. We focused on single-nucleotide polymorphisms (SNPs) occurring in the IL-13 promoter region, particularly IL13-1055C/T, IL13-591A/G, and IL13-1258A/G, where we observed interesting trends with regard to participants’ infection status in association with the polymorphisms they presented with. Also, we assessed their immune profiles after stratifying by age, and herein indicate trends which reflect observed age-associated trends between *S*. *haematobium* infection and the IL-13 polymorphisms. The outcomes presented indicate a need for further work which challenges the current ‘one size fits all’ strategy in mass chemotherapy programmes and advocates for participant-specific/sub-group tailored programmes. This is more likely to minimize the likelihood for the development of resistant *Schistosoma* strains to PZQ.

## Introduction

Schistosomiasis is a neglected tropical disease with a global prevalence second only to malaria [1]. Approximately two hundred and forty million people are actively infected worldwide, with the annual number of deaths ranging between 250,000 and 300,000 [2]; whilst about seven hundred million are at risk of infection [3,4]. Most of those infected live in resource-limited areas in sub-Saharan Africa [5,6]. Infections in humans occur when the bare skin comes in contact with slow-moving water bodies infested with free-swimming cercariae. Upon penetration by the cercariae, further larval development occurs during circulation in the human definitive host, culminating in the adult male and female schistosomes pairing up and settling inside the portal, mesenteric or pelvic veins where mating occurs. The female worms lay numerous eggs, many of which are trapped permanently in host tissues [7], with the rest extruded through urine (with respect to *S*. *haematobium*) to the external environment.

It is quite apparent from recent studies that, apart from climate and behaviour, other factors such as the individual’s immunity, age, and the host’s genetic make-up contribute to variations in infection levels [8–11]. Beyond the natural immune resistance to schistosomiasis, which develops after many years of repeated infection, there is also growing immuno-epidemiological evidence suggesting genetic associations with human resistance and susceptibility to schistosomiasis. The region of interest on the human genome is a locus, 5q31 –q33, which contains several genes related to immune function, including the T-helper 2 (Th2) cluster of genes, interleukin-4 (IL-4), interleukin-5 (IL-5), and interleukin-13 (IL-13) [12–14]. These cause the production of the cytokines IL-4, IL-5, and IL-13, which in turn influence Immunoglobulin E (IgE) levels and eosinophilia [1,13,15], components of immune response associated with infection/re-infection resistance in schistosomiasis [16–22].

Some studies have assessed the association of single nucleotide polymorphisms (SNPs) in the Th2 cluster of genes (i.e. IL-4, IL-5, and IL-13) with degree of susceptibility to schistosomiasis infections with varied objectives, and hence, outcomes. For instance, Kouriba and colleagues [13] sought to determine whether these SNPs were hereditary. Particular focus was therefore given to participants in their study population with high infection levels, and their parents. Along with providing evidence for some degree of SNP inheritability, they also acknowledged that IL13-1055T/T was associated with protection against schistosomiasis, as compared to IL13-1055C/T and IL13-1055C/C. Ellis *et al*. [23], in a nested case-control study conducted in China among 779 individuals aged 25 years and above with extensive exposure to infection, found no significant association between degree of susceptibility to *S*. *japonicum* re-infection and any of the SNPs studied in the IL-4, IL-5,and Il-13 genes. Moreover, two SNPs in the IL-5 and IL-13 genes, which appeared to be associated with infection susceptibility, had to be considered as likely due to chance, because both associations were no longer significant upon permutation testing. In a longitudinal study involving Kenyan men occupationally exposed to schistosomiasis-infested water bodies, Gatlin and team [22] sought to assess the level of synergy between the Th2 cluster of genes and the IFN-g, as well as IL-10, genes, in conferring resistance to re-infection after treatment. Outcomes from their work indicated on one hand, a synergistic association between the heterozygous IL13-1055C/T and the homozygous IFN-g +874A/A genotypes in conferring resistance to *S*. *mansoni* re-infection. On the other hand, homozygous IL13-1055C/C or IL13-1055T/T in association with heterozygous IFN-g +874A/T was found to increase resistance to re-infection; whilst in a third scenario, heterozygous IL4-590C/T was strongly associated with resistance to re-infection.

Given the varied outcomes of these and other studies, as well as the apparent paucity of data on school-aged children (SAC) who present with the highest risk of infection/re-infection to schistosomiasis, we sought in this cross-sectional work, to assess the associations between SNPs in the IL-13 gene promoter region (namely, IL13-1055C/T, IL13-591A/G and IL13-1258A/G) and schistosomiasis infection levels among selected schoolchildren in four *S*. *haematobium*-endemic communities.

In order to assess the expressions of protective and suppressory immune responses among selected pupils, titres of *S*. *haematobium* soluble egg antigen (ShSEA)-specific IgG and IgE, as well as total IgA, IgE, IgG1, and IgG4 were also measured. This was due to the influence of ShSEA-IgG and–IgE on egg production of the Schistosome [24]; the association of total IgA with protective immunity at human host mucosal surfaces [25,26]; total IgE and IgG1 with increased resistance to *S*. *haematobium* re-infection [24,25]; and total IgG4 with immune regulation and reduced resistance to re-infection [24]. Additionally, we measured the T-helper 2 (Th2) cytokines interleukin 4 (IL-4), IL-5, and IL-13, given their role in inducing anti-helminth immune responses [27]; as well as IL-10, an immune regulatory cytokine, and a precursor of IgG4 production [28,29].

Findings are expected to strengthen the current body of evidence pointing towards the need to modify current mass drug administration strategies to ensure greater focus on participants at higher risk of infection/re-infection.

## Materials and methods

### Ethics statement

Approval for the study was granted by the Ghana Health Service Ethical Review Board (GHS-ERB). Additional clearance was obtained from regional and district education directors, as well as from community leaders, and school administrators. The study was explained to parents/guardians, and pupils in durbars organized for the purpose. Written formal consent was obtained from interested parents/guardians on behalf of their wards; and written formal assent from the pupils.

### Study areas

The Ashanti Region (AR) is in the southern half of Ghana, with nearly three-quarters of it lying within the semi-equatorial forest zone, and the rest within the savanna zone. Two rainy seasons and a relatively short dry season, as well as an atmospheric temperature of 27°C, essentially characterize prevailing climatic conditions. The region is also endowed with lakes, waterfalls, rivers, among others [30]. The two study communities selected in this region were Asempaneye (N 06° 47’ 55.2”; W 001° 49’ 14.2”) and Barikuma (N 06° 49’ 28.4”; W 001° 45’ 26.0”). The Central Region (CR) extends from Ghana’s coastline and tapers inland, sharing boundaries with the AR and three other regions. The two rainfall seasons are shorter in duration, and climatic conditions less humid. Nevertheless, rivers and lakes are a characteristic feature of the region’s geography (Ghana Statistical Service, 2014). The study communities selected in this region were Brimsu Apewosika (N 05° 11’ 50.82”; W 001° 16’35.74 and Eyan Akotoguah (N 05° 19’ 23.9”; W 001° 02’ 36.1”) (S1A Fig in S1 Text).

### Study design

**Fig 1:**
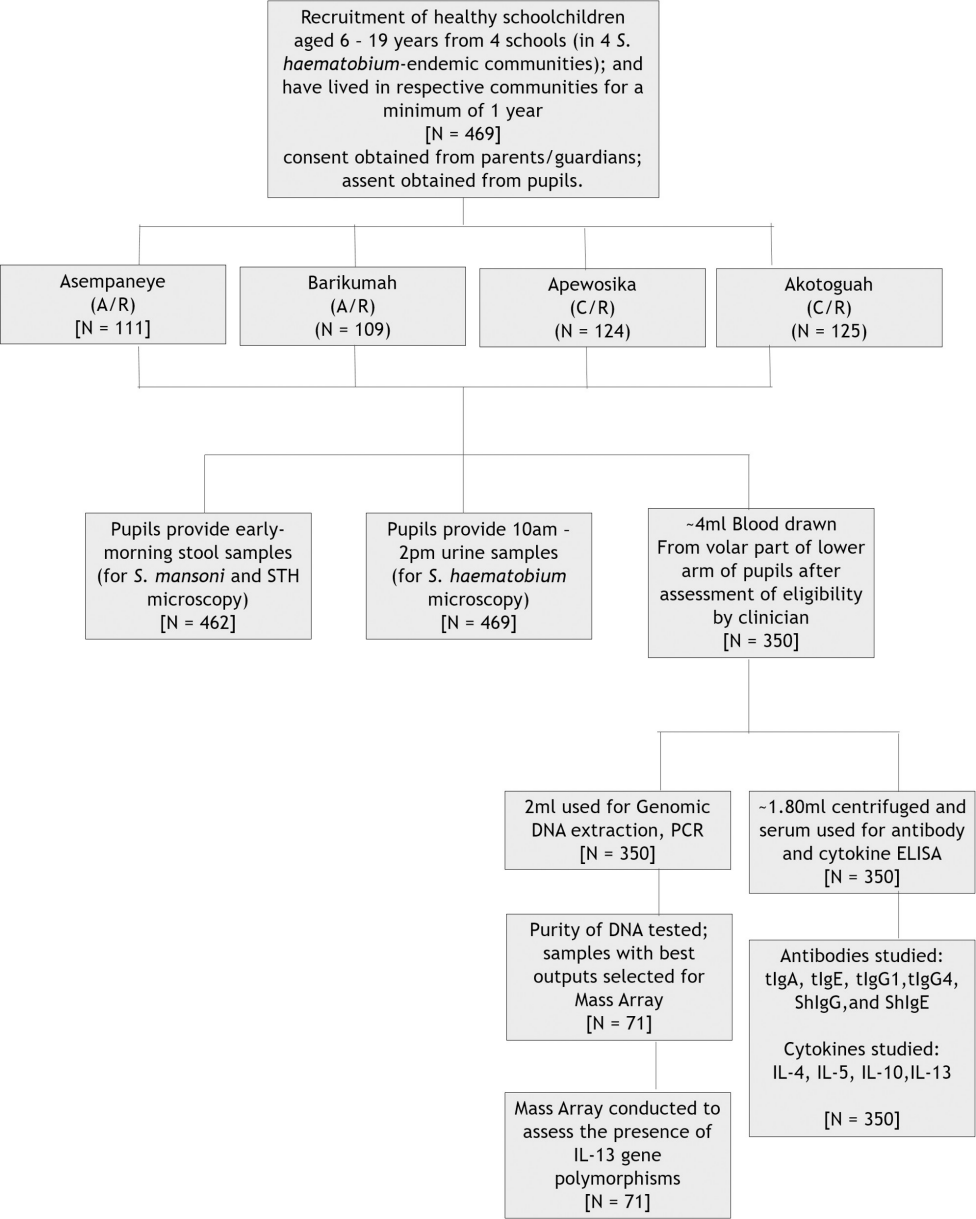
Flow chart of the study detailing the number of pupils involved at each phase (i.e. for each of the techniques employed). A/R denotes the Ashanti Region of Ghana; C/R represents the Central Region.

A total of 469 pupils aged 6 to 19 years were recruited (subsequent to obtaining signed/thumb-printed informed consent/assent) from a selected, government-run, basic school in each of the four study communities. Each participant presented with a single urine, and stool sample in respective containers provided. Willing and healthy participants (following assessment by a clinician) also donated 4mls of blood in ethylene diamine tetraascetic acid (EDTA) tubes. Urine and stool samples were processed using the sedimentation and Kato-katz techniques respectively, and examined for helminth eggs using microscopy. Blood samples were processed, and the levels of *S*. *haematobium*-soluble egg antigen (ShSEA)-specific IgG and IgE, as well as total IgG1, IgG4 and IgA, were measured using ELISA, which was also employed to determine the levels of the cytokines IL-4, IL-5, IL-10, and IL-13. Of the 350 blood samples donated from the 4 communities, 71 were randomly selected irrespective of infection status, for sequencing using the Mass Array technique, and the detection of IL-13 SNPs (Fig 1). In addition to studying the association between IL-13 and *S*. *haematobium* infection, the associations of co-factors and covariates with IL-13 SNPs, and with *S*. *haematobium* infection were assessed.

### Inclusion/exclusion criteria

Participants willing to donate blood were assessed on their eligibility through the assistance of a clinician, and via a short questionnaire. Only participants who had lived in the selected communities for at least a year; did not look pale or anaemic; and had no history of haemophilia, or of receiving blood transfusion prior to this work, were recruited.

### Urine

Urine samples were collected from each study participant into labelled 50mLfalcon tubes (BD Biosciences, Bedford, MA, USA), between 10.00 and 14.00 GMT, and transported to the laboratory on ice. Approximately 10ml was pipetted from each 50ml tube after homogenization (via gentle shaking) into sterile, respective 15ml falcon tubes (BD Falcon, USA), and centrifuged at 2000 revolutions per minute (rpm) for five minutes. The supernatant was discarded and the sediments transferred to a microscope slide and examined for *S*. *haematobium* eggs.

### Stool

Pupils were also given labelled containers for the submission of their early morning stool the following day. These were collected and transported to the laboratory before 08.00 hours GMT. Stool samples were processed for microscopy using the Kato-Katz kit with the 41.70 mg card template (Vestergaard Frandsen), as described elsewhere [31,32]. Two slides were prepared per sample and examined using microscopy for the ova of S. *mansoni*, the hookworms (*Ancylosotoma duodenale* and *Necator americanus*) and other STHs (*Ascaris lumbricoides*, and *Trichuris trichiura*). For each helminth species, the mean egg count from the two slides, was multiplied by a factor of 24 and expressed as eggs per gram (epg) of stool.

### Blood

Approximately 4ml of venous blood was drawn from the volar part of the lower arm of each subject into EDTA tubes and transported to the laboratory on ice. Samples were centrifuged at 3,400rpm for 10 minutes at room temperature (RT), and the plasma transferred with Pasteur pipettes into sterile 1.5ml Eppendorf tubes for storage at -80°C until needed.

### Preparation of crude *Schistosoma haematobium* egg antigens

Eggs obtained from *S*. *haematobium*-positive urine samples were utilized in an antigen preparation technique as previously described by Boros and Warren [33] with slight modifications. Briefly, egg-positive samples were centrifuged at 1200 rpm for 10 minutes at 4°C, and the supernatant discarded. Pellets were pooled into one 50mL falcon tube and washed twice with Phosphate Buffered Saline (PBS) at 1200 rpm for 10 minutes at 4°C. Sediments were homogenized on ice using a pre-chilled homogenizer (crucible) with a fitting pestle, and when approximately 95% of eggs had been disrupted (estimated by microscopic examination of preparation samples at intervals) the crude mixture was centrifuged at 2500 rpm at 4°C for 20 minutes. The supernatant was next centrifuged at 13000 rpm at 4°C for 60 minutes, and filtered using a 0.02μm filter. The eluate was aliquoted into 1.5mL Eppendorf tubes and stored at -20°C until ready to be used.

### Measurement of *S*. *haematobiu*m soluble egg antigen (ShSEA)-specific IgG (ShSEA-IgG) and IgE (ShSEA-IgE)

Measurement of plasma levels of *S*. *haematobium*-specific IgG and IgE was conducted using the indirect ELISA technique as described elsewhere [34]. Briefly, ninety-six well microtiter plates (Nunc Maxisorp, Thermofisher, USA) were coated with 100μL/well of crude *Schistosoma haematobium* soluble egg antigens (diluted 1000x from stock) in PBS and incubated overnight at 4°C. After washing thrice with PBS containing 0.05% Tween 20, the plates were blocked with PBS containing 3% skimmed milk for 1 hour and again washed. To each well was added 100uL of plasma from each participant, diluted in 1% milk/PBS (1:1000 for IgG measurement, and 1:200 for IgE measurement). On each plate were 7 standards, each in 3-fold serial dilutions, with starting dilutions of 1:100 (in PBS) for IgG, and 1:10 (in PBS) for IgE. The plates were sealed, incubated for 2 hours at room temperature, and then washed as earlier described. The addition of Horseradish peroxidase-conjugated goat anti-human IgG (diluted 1:1,000 in 1% milk/PBS) and IgE (diluted 1:500 in 1% milk/PBS) was next, and the plates again incubated at RT for 2 hours. After washing, 100μl of 3, 3’, 5, 5’-Tetramethylbenzidine (TMB) was added to each well, and the plates incubated in the dark at RT for 20 minutes. Reaction was stopped using sulfuric acid (H_2_SO_4_) and optical densities (OD) read at 450nm using an ELISA plate reader (Biotek Elx 808, USA). The ADAMSEL 4.0 software (Ed Ramarque, BPRC) was used to convert OD values into antibody units.

### Measurement of total IgA, IgE, IgG1 and IgG4 concentrations in plasma samples

Plasma levels of IgA, IgE, IgG1and IgG4 were measured using Ready-Set-Go ELISA kits (affymetrix eBioscience, USA), with strict adherence to the manufacturer’s protocols. Briefly, 96-well ELISA plates (provided by the manufacturer) were coated with 100μl/well of capture antibody (purified anti-human IgA/IgE/IgG1/IgG4 monoclonal antibody) at a dilution of 1:250 in coating buffer. The plates were incubated overnight at 4°C, and washed twice with 400 μl/well wash buffer (PBS, 0.05% Tween-20). Wells were blocked with 250μl of blocking buffer (PBS, 1% Tween 20, 10% Bovine Serum Albumin (BSA)); incubated at room temperature for two hours; and washed. Into each respective well was added 100 μl of plasma samples diluted in assay buffer (1:10,000, 1:10, 1:2,000 and 1:1,000 dilutions for IgA, IgE, IgG1 and IgG4 respectively), while 100μl of assay buffer only, was pipetted into each of the blank wells. Also, 2-fold serial dilutions in duplicates of the standards were added using 100μl of reconstituted standards (Recombinant human IgA/IgE/IgG1/IgG4 in assay buffer) to generate standard curves for the determination of sample antibody concentrations. The plates were incubated at room temperature for 2 hours, and washed. This was followed with the addition of 100μl of respective horse radish peroxidase (HRP)-conjugated anti-human IgA/IgE/IgG monoclonal antibody (1:250 dilution in assay buffer) to each well, after which plates were incubated at RT for 1 hour and washed. The addition of 100 μl per well of substrate solution (TMB) was followed with the incubation of plates in the dark at RT for 15 minutes, and the addition of 100μl/well of stop solution (0.2M H_2_SO_4_). The ODs of the wells and antibody concentrations were determined as earlier described.

### Determination of Concentrations of Plasma Cytokines (IL-4, IL-5, IL-10, IL13)

Plasma levels of human interleukins were measured using Ready-Set-Go ELISA kits (affymetrix eBioscience, USA), with strict adherence to the manufacturer’s protocol. Briefly, 96-well plates (provided by the manufacturer) were coated with100μL/well of capture antibody diluted 1:250 in coating buffer, and incubated overnight at 4°C. Plates were washed thrice with 250μL/well wash buffer, blocked for 1 hour with 200 μL/well blocking buffer (PBS, 1% BSA) at room temperature, and washed. A two-fold serial dilution each of the reconstituted standards in duplicates was prepared, and 100 μl of each preparation added to respective wells (Recombinant cytokine in 100μL of diluent). To blank wells were added 100μl of diluent only, while 100μL of undiluted samples were added to sample wells in duplicates. Plates were incubated at RT for 2 hours and washed, after which 100μl/well of diluted biotinylated detection antibody (1:250 in assay diluent) was added, and plates incubated at RT for one hour. After washing, 100 μL of diluted avidin-horseradish peroxidase (1:200 in assay diluent) was added, and plates incubated at RT for 30 minutes. Plates were next developed with 100μL/well of TMB in the dark for 15 minutes, and reactions stopped with 50μl/well of stop solution (0.2M H_2_SO_4_). Optical densities were read as earlier described.

### Genomic DNA extraction and polymerase chain reaction

Genomic DNA was extracted from EDTA blood samples using a blood DNA extraction kit (Bioline, Meridian Life Sciences, Memphis, TN, USA), and with strict adherence to the manufacturer’s protocol. Extracted DNA was stored at -20°C until needed.

Conventional polymerase chain reaction (PCR) was conducted in a 25μl reaction volume containing 2μl DNA template, 1x standard buffer, 1.5mM MgCl_2_, 0.3mM each of dATP, dCTP, dTTP, and dGTP; 200nM each of forward and reverse primers, and 1.25U Taq Polymerase. The primers used to amplify locus 591 of the IL-13 gene were 5’- CCA GCC TGG CCC AGT TAA GAG TTT-3’ (forward) and 5’- CTA ATT CCT CCT TGG CCC CAC T-3’ (reverse); those for amplifying locus 1055 of IL-13 were 5’-ATG CCT TGT GAG GAG GGT CAC-3’ (forward) and 5’- CCA GTC TCT GCA GGA TCA ACC-3’ (reverse); while those for amplifying locus 1258 of IL-13 were 5’- GGC CCT CTA CTA CAG ATT AGG AAA-3’ (forward) and 5’- CCG TCC TGT CCT CGA TGG GGC T-3’(reverse). Amplification was performed using a DNA Engine Peltier Thermal Cycler (Bio-Rad, Hercules, Ca, USA). The IL13-591 primer conditions were 94°C initial denaturation for 5 minutes, followed by 34 cycles of 94°C denaturation for 1 minute, 61°C annealing for 45 seconds, 72°C extension for 45 seconds, and 72°C final extension for 3 minutes. The IL13-1055 and -1258 primer conditions were 95°C initial denaturation for 3 minutes, followed with 30 cycles of 95°C denaturation for 30 seconds, 62°C annealing for 30 seconds, 72°C extension for 1 minute, and 72°C final extension for 3 minutes.

The PCR products were resolved in 2% Agarose gel and electrophoresed, using ethidium bromide (EtBr) as the fluorescent staining dye, bromophenol blue as a tracking dye and a 100/50 base-pair DNA ladder. The EtBr-stained gel was visualized in the BioDoc-IT Imaging System (Ultra-Violet Products Limited, Upland, Ca, USA), and the DNA ladder used to estimate and confirm expected band sizes of segments of interest.

### Genotyping of polymorphisms in interleukin genes

A subset of samples (irrespective of infection status) with good bands and appropriate concentration, were selected and sent to Inqaba Biotechnology Laboratory in Pretoria, South Africa; where they were genotyped by Mass Array Technology to determine polymorphisms in the promoter regions of the IL13 gene (S1B Fig in S1 Text).

### Statistical analyses

All data was entered in Microsoft Office Excel version 10 (Microsoft Corporation, USA), and exported to SPSS version 20 statistical software (IBM Corporation, USA) for further analysis. Graphs were prepared using the GraphPad Prism software version 5 (GraphPad, La Jolla CA, USA). The association between IL-13 gene polymorphisms and *S*. *haematobium* infections were tested with the null hypothesis that, the variations in the genotypes for IL13-1055C/T, IL13-591A/G, and IL13-1258A/G had no association with *S*. *haematobium* infection positivity; whilst the alternative hypothesis would suggest that variations in the genotypes for IL13-1055C/T, IL13-591A/G, and IL13-1258A/G was associated with *S*. *haematobium* infection positivity. *S*. *haematobium* infection prevalences between groups were assessed using the chi-square (χ^2^) test. Infection intensity comparisons between groups were done using either the Mann Whitney U (for two groups), or the Kruskal Wallis (for three groups or more) tests. Correlations between *S*. *haematobium* infection and immunology data was done using the Spearman’s rank correlation (Spearman’s rho), whilst comparisons of immunology data between genotype groups were done using either the independent t-test (for two groups), or the one-way analysis of variance (ANOVA), as immune measurements were parametric (from normality tests). Comparisons presenting with p-values less than 0.05 were deemed significant.

## Results

### Demographic characteristics of study participants

A total of 469 pupils were recruited from public schools in the four rural, *S*. *haematobium*-endemic communities, namely Apewosika (N = 124) and Akotoguah (N = 125) in the Central Region; and Barikumah (N = 109) and Asempaneye (N = 111) in the Ashanti Region of Ghana (Table 1). The ages of the schoolchildren ranged from 6 to 19 years, with a mean age of 12.20 years (±0.12 years), which did not vary significantly between the study communities (Table 1). Participants were further categorized into three age strata, namely, ≤11 years, 12 to 14 years, and 15 years or older. The largest age stratum with regard to number of participants was the 12 to 14-year age group. No significant differences were observed between the number of males and females involved in the study for both the pooled, and the study community datasets (Table 1).

**Table 1:** Characteristics of study participants.

Parameter	Pooled (N = 469)	Asempaneye (N = 111)	Akotoguah (N = 125)	Apewosika (N = 124)	Barikumah (N = 109)
Age in years (mean, SEM)	12.20 (±0.12)	12.00 (±0.26)	13.35 (±0.20)	11.45 (±0.24)	11.94 (±0.25)
Age range in years	6–19	6–19	8–19	6–17	6–17
Age Groups in years [n/N (%)]					
≤11	174/469 (37.10)	48/111 (43.24)	43/125 (34.40)	28/124 (22.58)	55/109 (50.45)
12–14	143/469 (84.62)	27/111 (24.32)	39/125 (31.20)	37/124 (29.84)	40/109 (36.69)
15+	152/469 (32.41)	36/111 (32.43)	27/125 (21.60)	60/124 (48.39)	29/109 (26.61)
p-val	0.379	0.252	0.618	0.138	0.644
Gender[(n/N)]					
Male	263/469 (56.08)	63/111 (56.76)	72/125 (57.60)	67/124 (54.03)	61/109 (55.96)
Female	206/469 (43.92)	48/111 (43.24)	53/125 (42.40)	57/124 (45.97)	48/109 (44.04)
p-val	0.95	0.155	0.089	0.37	0.213
Infection Prevalences					
*S*. *haematobium* [n/N, (%)]					
infected	99/469 (21.11)	19/111 (17.12)	26/125 (20.80)	19/124 (15.32)	35/109 (32.11)
uninfected	370/469 (78.89)	92/111 (82.88)	99/125 (79.20)	105/124 (84.68)	74/109 (67.89)
*S*. *mansoni* [n/N (%)]					
infected	5/469 (1.07)	2/107 (1.87)	3/125 (2.40)	0/124 (0.00)	0/109 (0.00)
uninfected	457/469 (97.44)	105/107 (98.13)	122/125 (97.60)	124/124 (100.00)	109/109 (100.00)
STH [n/N (%)]^§^					
infected	47/469 (10.02)	17/111 (15.32)	3/125 (2.40)	13/124 (10.48)	14/109 (12.84)
uninfected	422/469 (89.98)	94/111 (84.68)	122/125 (97.60)	111/124 (89.52)	95/109 (87.16)
Selections for Mass Array studies	71	18	17	15	21
Immunology [Mean (min-max)]					
log^*k*^ ShIgE (ug/ml)^*a*^	0.24 (0.01–1.27)	0.25 (0.01–1.27)	0.27 (0.4–0.96)	0.27 (0.04–0.96)	0.20 (0.02–0.44)
log ShIgG (ug/ml)^*b*^	2.54 (1.09–3.57)	2.55 (1.82–3.10)	2.54 (1.82–3.75)	2.48 (1.09–3.75)	2.54 (2.08–2.92)
log tIgG1 (ug/ml)^*c*^	1.15 (0.59–2.09)	1.01 (0.59–1.42)	1.26 (0.83–2.09)	1.12 (0.59–1.65)	1.21 (0.66–1.74)
log tIgG4 (ug/ml)^*d*^	2.35 (0.95–2.85)	2.36 (2.06–2.71)	2.51 (1.54–2.77)	2.36 (1.10–2.85)	2.19 (0.95–2.73)
log tIgE (ug/ml)^*e*^	0.39 (0.04–1.11)	0.38 (0.08–0.88)	0.75 (0.26–0.88)	0.34 (0.04–1.11)	0.37 (0.11–0.85)
log tIgA (ug/ml)^*f*^	3.59 (2.07–5.30)	3.47 (2.07–5.30)	3.65 (3.02–4.49)	3.49 (2.51–4.69)	3.77 (2.72–5.04)
log IL-4 (pg/ml))^*g*^	0.39 (0.11–0.95)	0.30 (0.11–0.51)	0.32 (0.20–0.67)	0.46 (0.11–0.95)	0.44 (0.11–0.86)
log IL-5 (pg/ml)^*h*^	0.76 (0.30–4.05)	0.74 (0.30–1.78)	0.39 (0.30–1.44)	0.67 (0.30–1.60)	0.79 (0.30–4.05)
log Il-10 (pg/ml)^*i*^	1.60 (0.91–2.05)	1.61 (1.25–1.91)	1.59 (0.95–1.95)	1.60 (0.91–2.04)	1.58 (0.93–2.05)

**^§^**Soil-transmitted Helminths represent *Ascaris lumbricoides*, and Hookworm (*Ancylostoma duodenale*, *Necator americanus*).

*^a^* ShIgE–*Schistosoma haematobium*-specific immunoglobulin (Ig) E

*^b^* ShIgG–*Schistosoma haematobium*-specific IgG

*^c^* tIgG1 –total IgG1

*^d^* tIgG4 –total IgG4

*^e^* tIgE–total IgE

*^f^* tIgA–total IgA

*^g^* IL-4 –Interleukin-4

*^h^* IL-5 –Interleukin-5

*^i^*IL-10 –Interleukin-10

*^j^* IL-13 –Interleukin-13

*^k^* log–Antibody and interleukin data were converted to log_10_ values prior to analysis.

P-values between groups were determined using the Mann Whitney U and Kruskal Wallis tests. P-values less than 0.05 are in boldface.

### Prevalence of Helminth infections

With respect to *Schistosoma haematobium* infection, an overall prevalence of 21.11% was observed, representing 15.32% prevalence in Apewosika, 20.80% in Akotoguah (both in the Central Region); 17.12% in Asempaneye, and 32.11% in Barikumah (both in the Ashanti Region). The total prevalence of *S*. *mansoni* was however 1.07%, with community-level prevalences ranging from 0.00% (in Apewosika) to 2.40% (in Akotoguah) (Table 1). Total soil-transmitted helminth (STH) (*Ascaris lumbricoides*, and, Hookworm (*Necator americanus* and *Ancylosotoma duodenale*)) prevalence was 10.02%, and ranged from 2.40% to 15.32% for the communities under scrutiny (Table 1).

### Association of *Schistosoma haematobium* infection with age and gender

**Fig 2:**
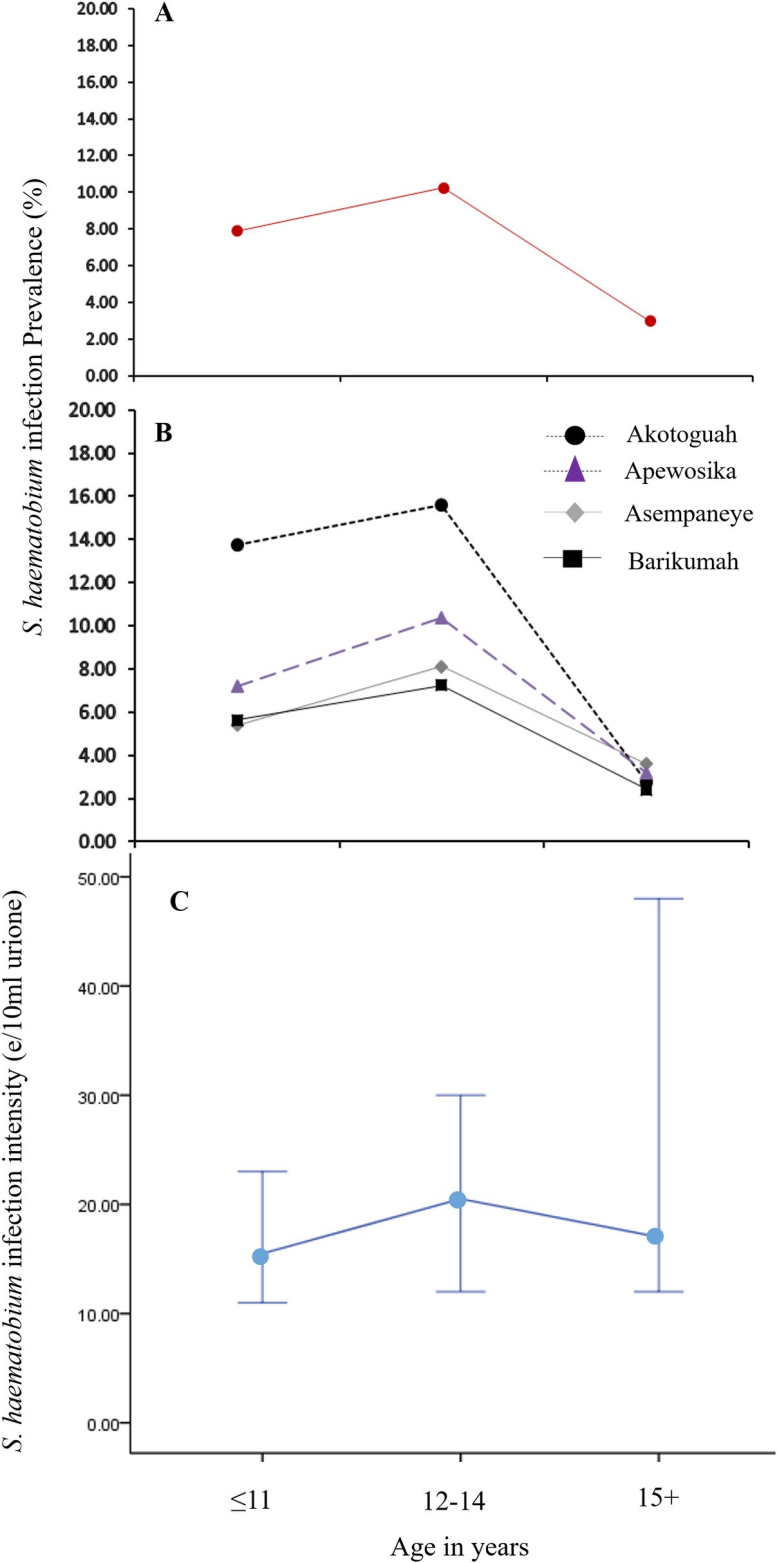
(A) Overall percent *S*. *haematobium* infection prevalence distribution by age groups (B) Percent S. haematobium infection prevalence distribution by age groups for the four study communities. (C) The overall *S*. *haematobium* infection intensity distribution by age groups.

The prevalence of *S*. *haematobium* infection among the 469 pupils was highest among those aged 12–14 years; and lowest among schoolchildren aged 15 years and above. This was also consistent for the community datasets (Fig 2). With regard to *S*. *haematobium* infection intensity, a similar trend was observed whereby geometric mean (GM) infection intensities increased with age, peaking at the 12-14-year age group, and then declining with further increase in age (Fig 2). Overall, higher GM *S*. *haematobium* infection intensity, and prevalence, were observed among recruited male than female pupils, although not significant.

### Association of IL-13 genetic polymorphisms with age, gender, *S*. *haematobium* and STH infections

Genomic DNA extraction and conventional PCR were conducted on 350 blood samples, of which 71, which presented with good bands (following gel electrophoresis; and irrespective of corresponding infection status), were selected for IL-13 gene polymorphism analyses (i.e. IL 13-1055C/T, IL 13-591A/G, and IL 13-1258A/G) by Mass Array. The distribution of 71 selected samples among the four study communities are presented in Table 1. Analyses of gene polymorphism frequencies as an assessment of adherence to the Hardy-Weinberg Equilibrium indicated no significant differences between expected and observed frequencies of the genotypes in the study population (Table 2).

**Table 2:** Determination of IL-13 gene polymorphism adherence to the Hardy-Weinberg Principle.

Polymorphisms	Genotypes	Frequencies in population Expected Observed		Hardy-Weinberg Equilibrium Test χ^2^ ^ȿ^ (P-value)
IL 13-1055C/T	C/C	24	20.88	2.23 (0.49)
	C/T	29	35.25	
	T/T	18	14.88	
IL 13-591A/G	A/A	39	41.07	1.82 (0.56)
	A/G	30	25.86	
	G/G	02	04.07	
IL 13-1258A/G	A/A	31	30.45	0.08 (0.95)
	A/G	31	32.09	
	G/G	09	08.45	

**^§^** Expected values were calculated using formulae based on the Hardy-Weinberg principle equation: p^2^ +2pq +q^2^ = 1.

^ȿ^ The Hardy-Weinberg χ^2^ test for deviation formula was used in determining whether there was a deviation of genotype frequencies from the principle

Furthermore, the strength of association between the IL-13 gene polymorphisms and a number of *a priori* factors including age, gender, as well as *S*. *haematobium* and soil-transmitted helminth (STH) infections were assessed for the 71 pupils (Table 3). There was no statistically significant difference between the age strata for all the three Il-13 gene polymorphisms under consideration, namely IL 13-1055C/T (i.e. C/C, C/T, T/T), IL 13-591A/G (i.e. A/A, A/G, G/G), and IL 13-1258A/G (i.e. A/A, A/G, G/G). Nevertheless, higher frequencies of all three IL13-gene polymorphisms were observed among pupils in the 12-14-year age group (Table 3). This was particularly significant by way of trends with regard to IL13-1258A/G (p-val. trends = 0.006). No significant difference was observed between males and females for all the three IL-13 gene polymorphisms under scrutiny (Table 3).

**Table 3:** Association of IL-13 genetic polymorphisms with study factors^*a*^.

Factor	IL13-1055 (N = 71)			IL13-591 (N = 71)			IL13-1258 (N = 71)		
	C/C	C/T	T/T	A/A	A/G	G/G	A/A	A/G	G/G
Age in years [n (%)]									
≤11	8 (11.27)	8 (11.27)	2 (2.81)	12 (16.90)	21 (29.58)	6 (8.45)	8 (11.27)	18 (25.35)	5 (7.04)
12–14	14 (19.71)	11 (15.49)	4 (5.63)	17 (23.94)	10 (14.08)	3 (4.23)	15 (21.12)	12 (16.90)	4 (5.63)
15+	8 (11.27)	13 (18.31)	3 (4.23)	1 (1.41)	1 (1.41)	0 (0.00)	7 (9.86)	2 (2.82)	0 (0.00)
p-val	0.817			0.291			0.065 (p-val (trends) = **0.008**)		
Gender									
Male	13 (18.31)	15 (21.13)	8 (11.27)	19 (26.76)	15 (21.13)	2 (2.82)	17 (23.94)	15 (21.13)	4 (5.63)
Female	11 (15.49)	14 (19.72)	10 (14.08)	20 (28.17)	15 (21.13)	0 (0.00)	15 (21.13)	17 (23.94)	5 (7.04)
p-val	0.815			0.366			0.841		
*S*. *haematobium*									
infected	10 (14.08)	15 (21.13)	5 (7.04)	15 (21.13)	14 (19.72)	1 (1.41)	9 (12.68)	15 (21.13)	6 (8.45)
uninfected	14 (19.72)	14 (19.72)	13 (18.31)	24 (33.80)	16 (22.54)	1 (1.41)	23 (32.39)	17 (23.94)	3 (4.23)
p-val	0.27			0.772			0.078 (p-val (trends) = **0.025**)		
Soil-transmitted Helminths^*c*^									
infected	5 (7.04)	5 (7.04)	1 (1.41)	7 (9.86)	4 (5.63)	0 (0.00)	5 (7.04)	3 (4.23)	3 (4.23)
uninfected	19 (26.76)	24 (33.80)	17 (23.94)	32 (45.07)	26 (36.62)	2 (2.82)	27 (38.03)	29 (40.85)	6 (8.45)
p-val	0.378			0.721			0.206		

Comparisons between participants with the various groups were conducted using the χ^2^ tests.

*^a^* The pooled data was used in the analyses (i.e. where N = 71)

*^b^* Number of participants in the group of interest (percent prevalence or percent frequency)

*^c^*Soil-transmitted Helminths represent *Ascaris lumbricoides*, and Hookworm (*Ancylostoma duodenale*, *Necator americanus*).

**Fig 3:**
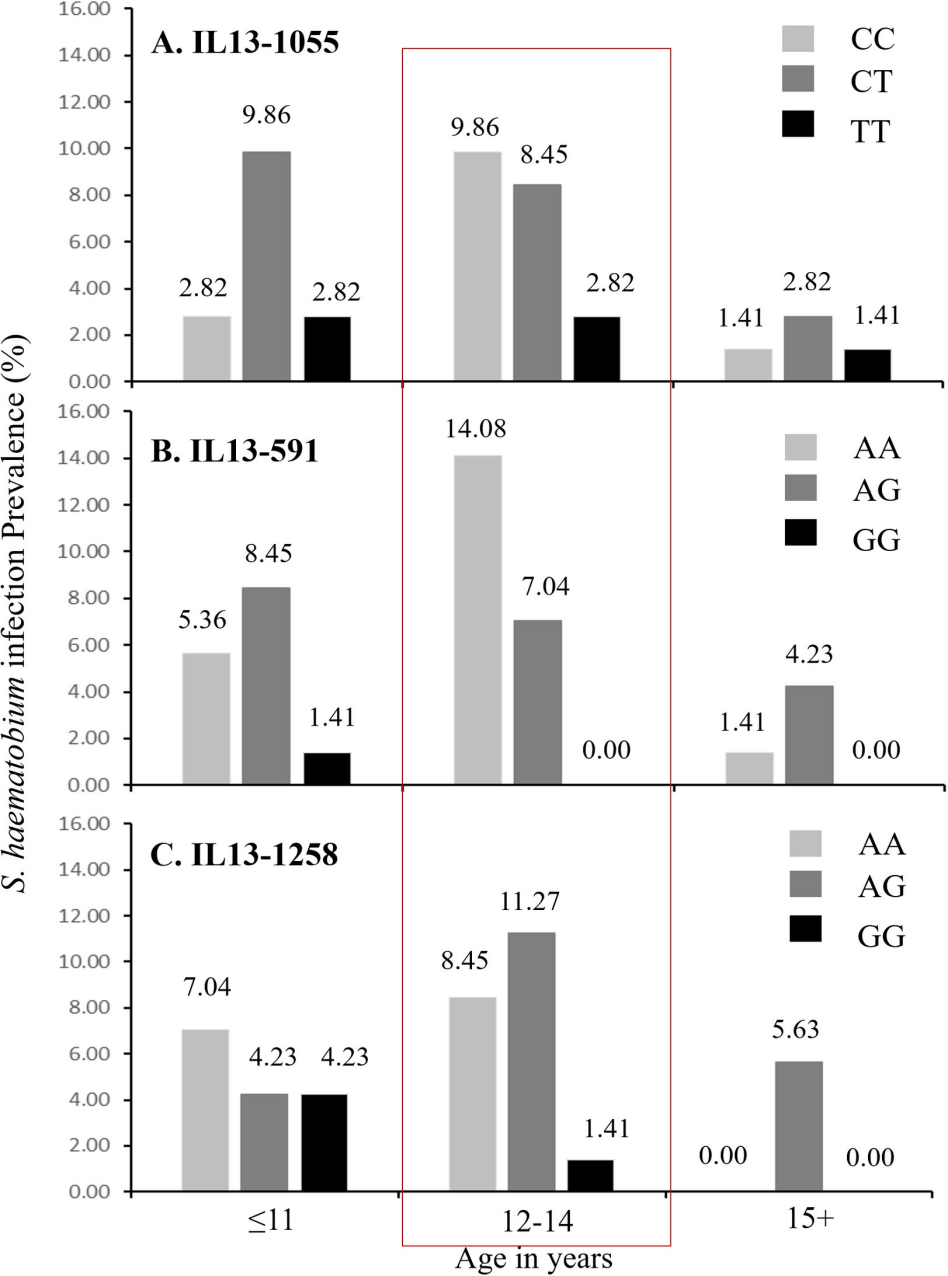
Percent *S*. *haematobium* infection prevalence distribution for (A) IL13-1055C/T, (B)IL13-591A/G, and (C)IL13-1258A/G polymorphisms by age groups. The red rectangular border highlights an observed infection prevalence distribution trend with the 12-14-year age group that is apparent for all three polymorphisms.

**Fig 4:**
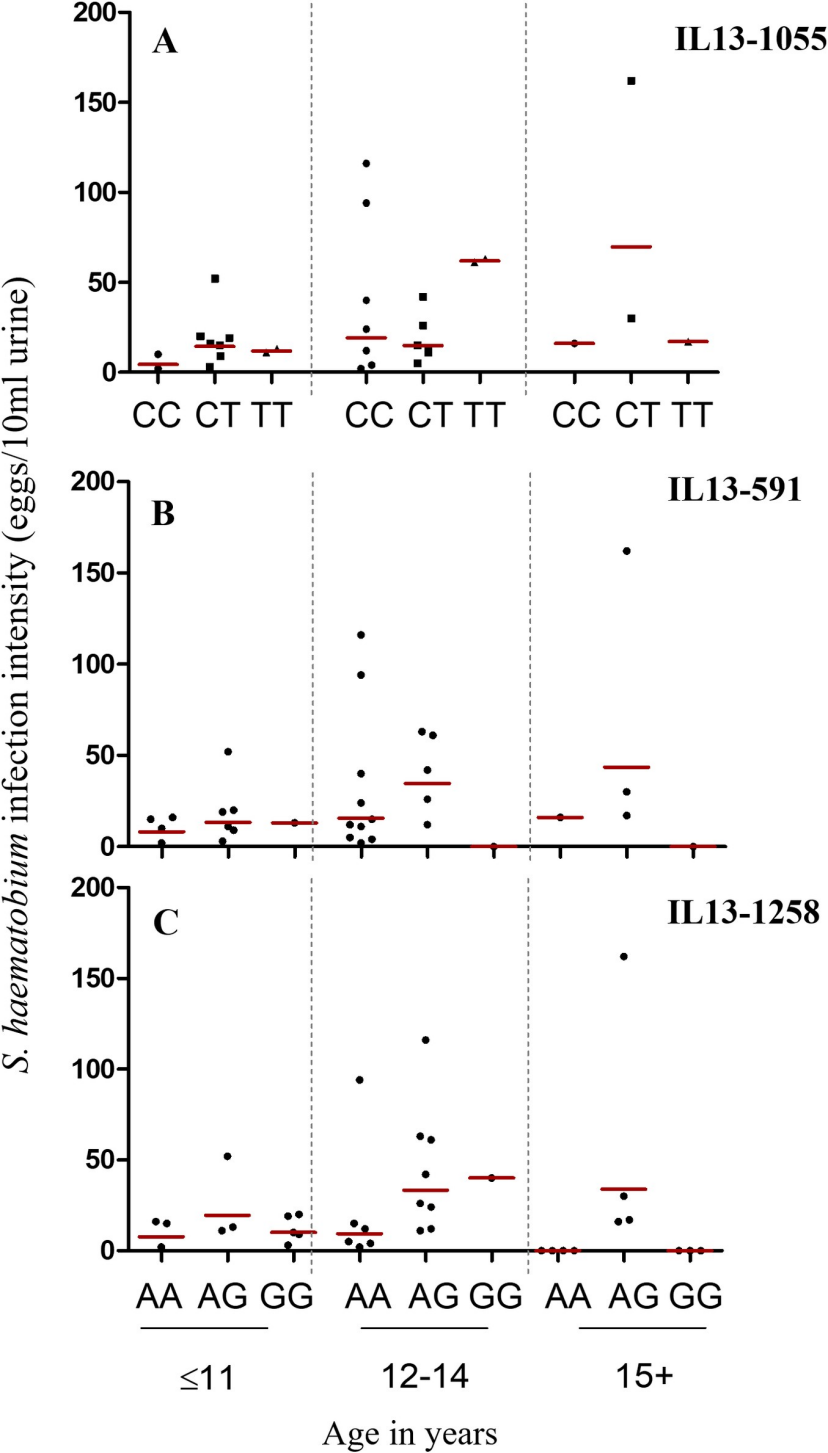
Geometric mean (GM) *S*. *haematobium* infection intensity distribution by Age for IL13-1055C/T, IL13-591A/G, and IL13-1258A/G polymorphisms. Red horizontal lines denote the GM intensity for the respective groups.

Furthermore, the highest percent prevalences of *S*. *haematobium* infection also occurred among pupils aged 12–14 years. Further stratification by gene polymorphisms indicated higher infection prevalences among pupils with the IL13-1055C/C or IL13-1055C/T; the IL13-591A/A or IL13591A/G; and the IL13-1258A/A or IL131258A/G genotypes, especially for the 12-14-year age category (Fig 3). In all, higher *S*. *haematobium* infection prevalences were observed for genotypes with the IL13-1055C, the IL13-591A, and the IL13-1258A (i.e. A/A and A/G; p-val trends = 0.025) alleles. Also, the lowest *S*. *haematobium* infection prevalence was observed among participants with genotypes homozygous for the IL13-1055T (i.e. T/T), IL13-591G (i.e. G/G), and the IL13-1258G (i.e. G/G) alleles (Table 3). Furthermore, higher geometric mean (GM) infection intensities were observed among pupils aged ≤11, and 15 years and above with the heterozygous genotype for IL13-1055C/T (i.e. C/T), IL13-591A/G (A/G), and for IL13-1258A/G (i.e. A/G) (Fig 4). However, higher GM infection intensities were realized among pupils aged 12–14 years who were homozygous for the T allele with regard to the IL13-1055C/T (i.e. T/T), and for the G allele with regard to the IL13-1258A/G (i.e. G/G) polymorphisms (Fig 4). With regard to STH infections, higher infection prevalences were realized among pupils with the IL13-1055C, IL13-591A, and the IL13-1258A alleles (Table 3).

### Immunological Profile of study participants and associations with *S*. *haematobium* infection

The immune factors measured for the 350 blood samples included the antibodies ShSEA-IgE, ShSEA-IgG (ShIgG), total IgG1 (tIgG1), total IgG4 (tIgG4), total IgE (tIgE), and total IgA (tIgA); as well as the Th2 cytokines interleukin-4 (IL-4), interleukin-5 (IL-5), interleukin-13 (IL-13); and the regulatory cytokine interleukin-10 (IL-10). For both the pooled and study community datasets, the levels of tIgA were observed to be the highest among the antibodies studied, whilst the levels of IL-10 were observed to be the highest among the cytokines studied (Table 1). All antibodies correlated negatively with *S*. *haematobium* infection intensity, except for tIgE (Spearman’s rho = 0.162, p = 0.260) and tIgA (Spearman’s rho = 0.214, p = 0.140) (S1C Fig in S1 Text). Apart from IL-5 (Spearman’s rho = 0.070; p = 0.662), all other cytokines also correlated negatively with *S*. *haematobium* infection intensity (S1D Fig in S1 Text).

The antibody and cytokine levels of *S*. *haematobium*-infected and -uninfected schoolchildren (N = 350) were compared. Generally, no significant difference with regard to mean antibody levels was observed between *S*. *haematobium*-infected and -uninfected pupils for the pooled dataset. Also with regard to the study cytokines, no significant differences were observed between infection groups (Table 1).

**Fig 5:**
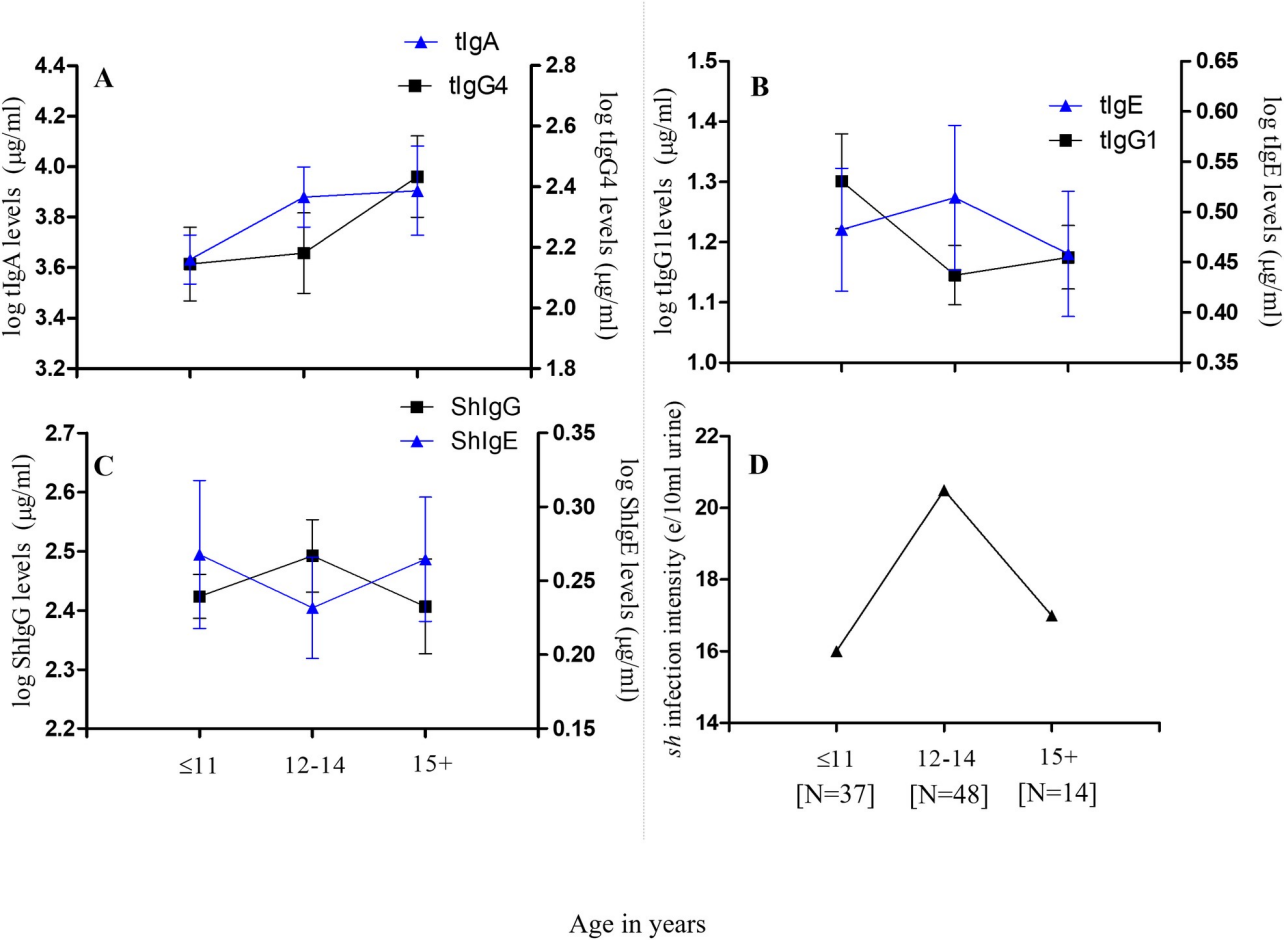
Stratification of measured plasma antibody levels by age among *S*. *haematobium*-infected participants (N = 99): (A) Mean total IgA (tIgA) and tIgG4 titres by age groups; (B) Mean titres of tIgE and tIgG1 by age groups. Error bars represent the standard error of mean (SEM). (C) Mean titres of *S*. *haematobium*-specific *Schistosoma* egg antigen (ShSEA)–IgE and ShSEA-specific IgG by age groups; and (D) GM *S*. *haematobium* infection intensity distribution by age groups among infected participants. [N] denotes the number of pupils in each age group.

**Fig 6:**
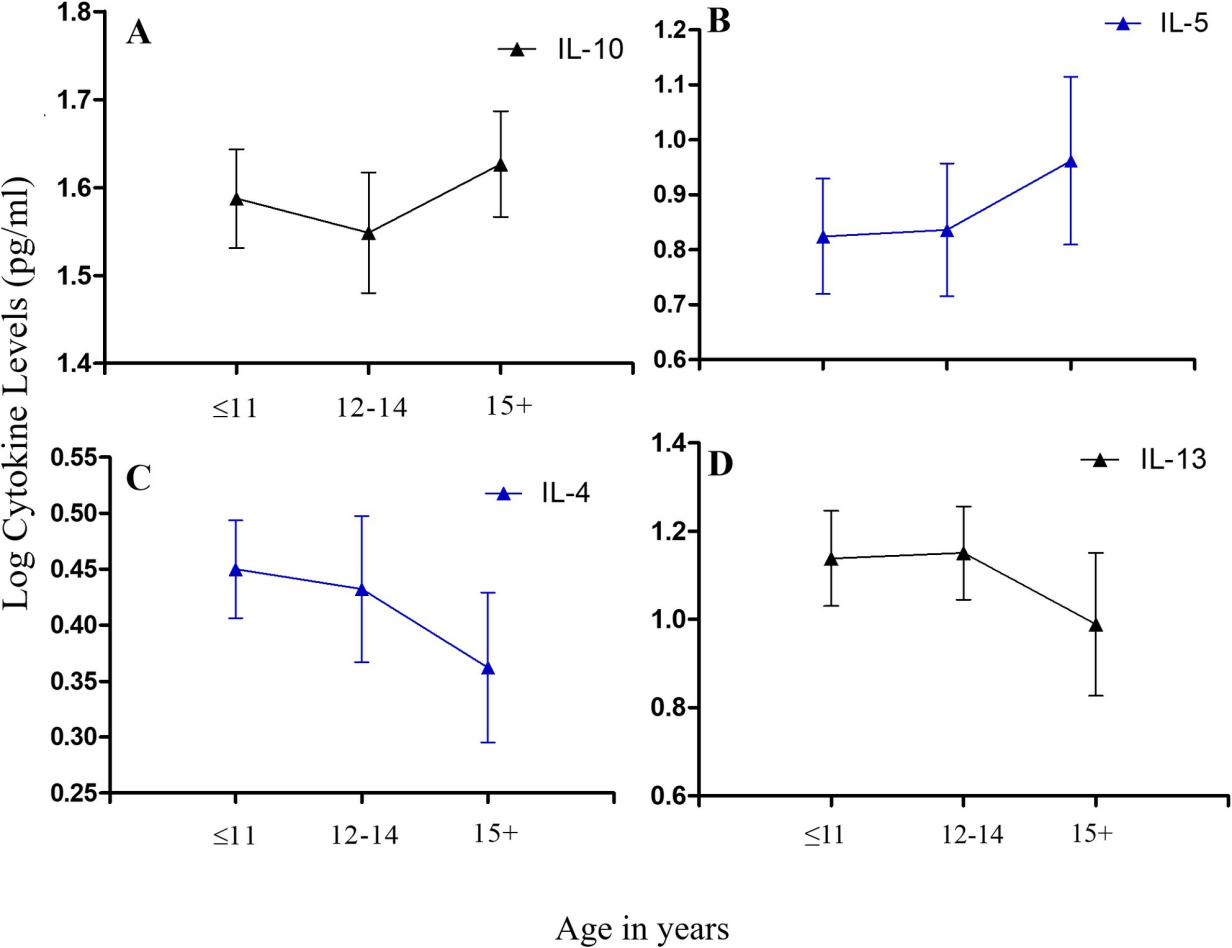
Stratification of measured plasma cytokine levels by age among *S*. *haematobium*-infected participants (N = 99): (A) Mean interleukin-10 (IL-10) titres by age groups; (B) Mean IL-5 titres by age groups; (C) Mean IL-4 titres by age groups and (D) Mean IL13 titres by age groups. Error bars represent the standard error of mean (SEM).

Assessment of immune responses was furthermore restricted to *S*. *haematobium*-infected pupils (N = 99). Stratifying these responses by age groups, we observed mean tIgA levels to increase with age, peaking among pupils aged 15 years or older. Similar trends were observed for tIgG4. Mean tIgE levels increased and peaked among schoolchildren aged 12–14 years, and then declined with further increase in age, while the opposite was apparent for tIgG1 titres. The age-associated trend for mean ShSEA-IgE titres was similar to that observed for tIgG1, while the age-associated trend for ShSEA-IgG appeared similar to that observed for tIgE (Fig 5). With regard to the cytokines, the age-associated trend observed among infected schoolchildren for mean IL-10 titres was similar to observations for tIgG4 and tIgA; while that of IL-13 showed striking similarity to that of tIgE. Mean IL-4 titres exhibited an age-associated trend similar to ShSEA-IgE, while the trend for IL-5 was oddly similar to those of tIgG4 and IL-10 (Fig 6).

### Association of IL-13 polymorphisms with immune responses

No significant variations were observed for the 71 participants, when antibody level and cytokine level comparisons were made with regard to the genotypes studied. In all however, tIgA levels were highest, followed with ShSEA-IgG, tIgG4, tIgG1, tIgE, and ShSEA-IgE levels. Among the cytokines, IL-10 presented with the highest levels, followed with IL13, IL4 and IL5. Additionally, higher (although insignificant) *S*. *haematobium* infection prevalence and intensity levels were observed among males than females. Assessing the association between STH infection status and *S*. *haematobium* infection revealed lower *S*. *haematobium* infection prevalence and intensities among participants who were STH-positive, as compared to those who were STH-negative.

## Discussion

This cross-sectional study was carried out with the primary aim of understanding the interplay between IL-13 gene polymorphisms and *Schistosoma* infections among schoolchildren in four schistosomiasis-endemic communities in the Central and Ashanti Regions of Ghana. Additionally, relationships between IL-13 gene polymorphisms and determined immune factors, such as antibody and interleukin concentrations in the blood of study participants, were assessed.

For the subset of 71 pupils, higher frequencies of genotypes bearing the alleles IL13-1055C, IL13-591A, and IL13-1258A, were observed among schoolchildren aged **≤**11 and 12–14 years. Interestingly, we found *S*. *haematobium* infection prevalence and intensity to be higher among pupils within these age strata whose genotypes carried the same alleles. Given the higher genetic frequency of these polymorphisms in the age groups, as compared to the homozygous T- or G-alleles among the selected participants, it is no surprise that this is the case. It could simply be indicative of a higher representation of the A- and C- alleles in this population. However, it would be highly beneficial that this trend is further assessed with larger cohorts in the same or other populations, as this may further explain why children 12 to14 years of age are often at the highest risk of *Schistosoma* infection, and often present with the highest levels of *Schistosoma* infection intensities in endemic areas. Findings from a number of studies indicate the C allele of the IL13-1055 polymorphism to be associated with a higher propensity to having *Schistosoma* infections. According to [13], the T allele, or the T/T genotype was indicative of a greater likelihood of protection from *Schistosoma* infections, whilst the reverse was true for the C/C genotype. Rahoud and team [35] also indicated the C allele, or the C/C and C/T genotypes to be associated with a higher risk in developing hepatic fibrosis in Sudanese patients; whilst He and colleagues in 2008 [15] showed through studying the STAT-6 gene (which is located in the IL-13 gene promoter) that, subjects with the C/T genotype were at a higher predisposition to *S*. *haematobium* infection. These findings also corroborate the study by Isnard and Chevillard [1], which showed higher infection levels for subjects with the C/C and C/T genotypes of the IL 13–1055 polymorphism. Additionally, studies conducted in the Boul and Segue communities in Mali showed higher *S*. *haematobium* infection intensities among participants with the C/C and C/T genotypes [13]. All these studies did indicate reduced *Schistosoma* infection to be associated with the T allele of the IL 13-1055C/T gene polymorphism. With respect to the IL 13-591A/G gene polymorphism, Kouriba and colleagues [13] found higher *S*. *haematobium* infection levels among participants with the A/A and A/G genotypes, than among participants with the G/G genotype. Information with respect to the IL 13-1258A/G gene polymorphism however, has been scanty. Nevertheless, evidence from this work indicate a similar trend in *Schistosoma* infection levels to those observed for the IL 13-591A/G gene polymorphism, in which *S*. *haematobium* infection was higher among participants with genotypes bearing the IL13-1258A allele. Infection intensity levels however appeared higher among pupils aged 12–14 years who were homozygous for the T and G alleles with regard to the IL13-1055C/T (i.e. T/T; N = 2) and IL13-1258A/G (i.e. G/G; N = 1) polymorphisms. Given however, the really small number of participants presented with such polymorphisms, it remains unclear whether this outcome accurately reflects what may prevail within this cohort of schoolchildren.

Furthermore, trends of association similar to those reported by Kouriba *et al*., [13], and by Isnard *et al*., [36] for *S*. *haematobium*, were observed between the IL-13 gene polymorphisms and soil-transmitted helminth (STH) infections. With regard to the IL13-1055 and IL13-591 polymorphisms, higher prevalences of STH infections were observed among pupils with genotypes bearing the IL13-1055C and the IL13-591A alleles, suggesting likely IL-13 gene polymorphism influence on STH and other helminth infection levels as well.

We observed *S*. *haematobium* infection intensity and prevalence for both the pooled and community data sets, to increase with age, peaking at the 12-14-year age group, and decreasing with further increase in age. A similar trend of infection was realized in a fishing community in Uganda, although for *S*. *mansoni* [37]. In Ghana, Aryeetey *et al*., [38], following studies conducted in eight communities in the Ga and Akuapem South districts, reported an *S*. *haematobium* infection intensity trend in which levels peaked among participants in the 12-14-year age group, and decreased with further increase in age. Asuming-Brempong *et al*., [39], also documented a similar age-associated trend of *S*. *haematobium* infection in a peri—urban community in the Eastern Region of Ghana.

With respect to gender, more males were documented in this study to have higher (although not statistically significant) levels of *S*. *haematobium* infection. Our observations of human activity at water contact sites indicated higher water contact activity, particularly swimming, among the male school children of our study communities. Gender differences with regard to *S*. *haematobium* infections were explained by Aryeetey and colleagues [38] to be due to differences in water-contact activity. We are unable to conclude in this regard, as water contact frequency was not actively measured among our participants. Differences in *Schistosoma* infection levels between males and females may therefore require more extensive immuno-epidemiological studies incorporating such factors as the cultural backgrounds of study cohorts, geography and climate, among others.

Considering age-associated immune responses among infected participants, we observed mean tIgA levels to increase with increasing age among our pupils. Interestingly, similar trends were observed for tIgG4 and IL-10, well-established factors of an immuno-regulatory response. Classically, IgA is said to neutralize toxins and microorganisms at mucosal/epithelial surfaces [40,41]. More recently however, IgA has been observed to protect from allergic inflammation via the neutralization of allergens in the mucosal lumen [26], and the inhibition of effector function of inflammatory cells [42]. Given that the major cytokine crucial for B-cells to class-switch to IgA production is TGF-β (a suppressory cytokine) [26,43], it is no surprise that similar age-associated trends were observed for tIgA, as for IL-10 and tIgG4 among our schoolchildren. On the contrary, mean tIgE, IL-13, and IL-4 levels were similar for pupils aged up to 14 years. Titres then declined steadily with increasing age. With regard to ShSEA-IgE, titres were highest among pupils aged 11 years or less, declined for pupils aged 12 to 14 years; and increased with further increase in age. With the exception of IL-5, all Th2 cytokines progressively declined with age. This is important, as these trends depict the immune profile of school-aged children in endemic, treatment-naïve communities. Indeed, the only study site in our selection which had undergone mass treatment was Akotoguah, and this was a year and six months before the commencement of this work. Thus, our data suggests that, schistosomiasis-infected school-aged children in a treatment-naïve setting at the age of 11 years or below, are likely to have high titres of both suppressory and inflammatory immune factors (most likely acquired from birth [44,45], as well as from exposure to environmental microorganisms.

As also indicated elsewhere [46], this is depictive of the well-characterized adaptive responses in schistosomiasis infections: the increasing pro-inflammatory immune environment associated with acute schistosomiasis infection, as shown in schoolchildren aged 11 years or less; and the increasing suppressive, anti-inflammatory immune environment associated with chronic infection as depicted in pupils aged 12 years or older. This also likely reflects observed *S*. *haematobium* infection trends with regard to IL-13 polymorphisms. The subsequent decrease in infection intensity among participants 15 years and older may be due to the combined influences of the immune response and participant behavior [47]. Interestingly, Mutapi and colleagues [48] found in serum samples of their study participants, high mean IgA levels which increased in *S*. *haematobium* (*Sh*)-infected children aged 5 to 16, and decreased considerably for Sh-infected participants aged 17 years and above; with the inverse observed for IgG1. This apparent contradiction to our observations may be due to the fact that, we measured total IgA and total IgG1, while they measured *S*. *haematobium* egg antigen (SEA)–specific IgA and–IgG1. Indeed, outcomes presented later by Mutapi *et al*. [46], from whole blood stimulation with a plant mitogen (Concanavalin A (Con A)), indicated trends for mean IL-4, IL-5, and IL-10 titres, which were similar to trends we observed for the same cytokines. This is important, as Con A generally stimulates T- and B-cell activity, whilst stimulation with *S*. *haematobium* antigens would likely induce more specific responses.

This study was cross-sectional, which is an important limitation, as data for only a particular time point could be acquired. Most immuno-epidemiological studies conducted to investigate the association of these cytokines with resistance to helminth infection are often longitudinal in nature, with a component involving treatment with PZQ. Nevertheless, we noted a negative correlation between IL-4 (as well as IL-13) and *S*. *haematobium* infection intensity. Another key limitation encountered was the number of participants involved in the IL-13 gene polymorphism component of the work, which was due partly to budgetary constraints. As a result, it was impossible, with the small numbers, to conduct model analyses to meaningfully assess the influencing role of other risk/environmental factors to participants’ infection with schistosomiasis. A third important limitation was the process of preparing crude *S*. *haematobium* antigens for this study, which did not include a Trichloroacetic acid (TCA) treatment step meant to improve sensitivity and remove any cross-reactive human antigens that may have attached while the egg was in the human host. As a result, some cross-reactivity may have unavoidably occurred. Although this was accounted for in determining threshold values, caution may still be necessary in the interpretation of immunology data presented. A fourth limitation involved the collection of single stool and urine samples per participant. It is acknowledged that in studies of this nature, a minimum of 2 samples are often collected on consecutive days. Due however to time, numeric and financial constraints, this was not done. We however prepared 2 examination slides per sample, and also performed quality assurance tests on every tenth slide prepared. This was done by highly qualified and well-experienced technicians.

In summary, this work makes the following submissions: firstly, that the genotypes carrying the IL 13-1055C, IL13-591A, and IL13-1258A alleles, appear more strongly associated with *S*. *haematobium* infection positivity and intensity; whilst the genotypes homozygous for the IL 13-1055T, IL 13-591G, and IL 13-1258G alleles (i.e. IL13-1055TT, IL13-591GG, and IL13-1258GG), may be associated with a reduced schistosomiasis infection. Second, the age-associated trends of measured antibodies and cytokines are indicative of the immune profiles of the schoolchildren in the treatment-naïve, *S*. *haematobium*-endemic study areas, and reflects observed *S*. *haematobium* infection intensity trends as well as IL-13 gene polymorphism patterns. Nevertheless, a longitudinal study with a treatment component and a larger cohort will be essential to more accurately assess the associations between immune factors and IL-13 gene polymorphisms.

## Supporting information

S1 Text **S1A Fig:** A map of the Central and Ashanti Regions, indicating the locations of the study communities. Map was developed by co-author using the QGIS Girona version 3.0.3 (Boston, MA., USA). Shape files of the regions of Ghana were obtained online (URL: https://github.com/tierney/gis-sandbox/tree/master/data/GIS-Ghana/ghana.shapefiles). Also, GPS data were obtained from the field, using appropriate devices, and exported to Microsoft Office Excel version 2013, where conversions were made. The document was then exported to the QGIS Girona version 3.0.3 software as a delimited text file. **S1B Fig:** Nucleotide sequences of the targeted sections of the IL-13 gene namely, (A) IL13-1055; (B) IL13-591; and (C) IL13-1258. Forward and reverse primer sequences for each of the targeted sections are in boldface. The target nucleotide for the single nucleotide polymorphism (SNP) in each section is underlined and red-lettered. **S1C Fig:** Box and whisker plots depicting overall median *S*. *haematobium* infection intensity distribution for IL13-1055C/T, IL13-591A/G, and IL13-1258A/G polymorphisms. Comparisons between groups were done using the Kruskal-Wallis and Dunn’s post tests. Mid-horizontal line denotes median infection intensities. Upper and lower whiskers denote 95^th^ and 5^th^ percentiles respectively. ‘*’ denotes p-values < 0.05. **S1D Fig:** Correlations of (A) ShIgG, (B) ShIgE, (C) tIgE, (D) tIgG1, (E) tIgG4, and (F) tIgA with S. haematobium infection intensity. ‘r’ denotes the Spearman’s rank correlation coefficient (Spearman’s rho). **S1E Fig:** Correlations of (A) IL-4, (B) IL-5, (C) IL-10, and (D) IL-13 with *S*. *haematobium* infection intensity. ‘r’ denotes the Spearman’s rank correlation coefficient (Spearman’s rho). Significant correlations are in boldface. **S1F Fig:** Stratification of measured plasma levels of (A & B) anti-inflammatory immune factors; and (C &D) pro-inflammatory immune factors by age groups. (A) Mean IL-10 and tIgG4 titres by age groups; (B) mean tIgA and IL-10 titres by age groups; (C) mean IL-13, tIgE, and ShIgE titres by age groups; and (D) mean IL-4 and ShIgE titres by age groups. Error bars represent the standard error of mean (SEM).
(DOCX)

S1 Data SPSS version of dataset used in analysis. (XLSX)
