# Supplementary material for: Associations of IL13 gene polymorphisms and immune factors with Schistosoma haematobium infection in schoolchildren in four schistosomiasis-endemic communities in Ghana
Source: PLoS Negl Trop Dis. 2021 Jun 29;15(6):e0009455. doi: 10.1371/journal.pntd.0009455 (PMC8274844; doi:10.1371/journal.pntd.0009455)
Supplement: S1 Text — S1A Fig: A map of the Central and Ashanti Regions, indicating the locations of the study communities. Map was developed by co-author using the QGIS Girona version 3.0.3 (Boston, MA., USA). Shape files of the regions of Ghana were obtained online (URL: https://github.com/tierney/gis-sandbox/tree/master/data/GIS-Ghana/ghana.shapefiles). Also, GPS data were obtained from the field, using appropriate devices, and exported to Microsoft Office Excel version 2013, where conversions were made. The document was then exported to the QGIS Girona version 3.0.3 software as a delimited text file. S1B Fig: Nucleotide sequences of the targeted sections of the IL-13 gene namely, (A) IL13-1055; (B) IL13-591; and (C) IL13-1258. Forward and reverse primer sequences for each of the targeted sections are in boldface. The target nucleotide for the single nucleotide polymorphism (SNP) in each section is underlined and red-lettered. S1C Fig: Box and whisker plots depicting overall median S. haematobium infection intensity distribution for IL13-1055C/T, IL13-591A/G, and IL13-1258A/G polymorphisms. Comparisons between groups were done using the Kruskal-Wallis and Dunn’s post tests. Mid-horizontal line denotes median infection intensities. Upper and lower whiskers denote 95th and 5th percentiles respectively. ‘*’ denotes p-values < 0.05. S1D Fig: Correlations of (A) ShIgG, (B) ShIgE, (C) tIgE, (D) tIgG1, (E) tIgG4, and (F) tIgA with S. haematobium infection intensity. ‘r’ denotes the Spearman’s rank correlation coefficient (Spearman’s rho). S1E Fig: Correlations of (A) IL-4, (B) IL-5, (C) IL-10, and (D) IL-13 with S. haematobium infection intensity. ‘r’ denotes the Spearman’s rank correlation coefficient (Spearman’s rho). Significant correlations are in boldface. S1F Fig: Stratification of measured plasma levels of (A & B) anti-inflammatory immune factors; and (C &D) pro-inflammatory immune factors by age groups. (A) Mean IL-10 and tIgG4 titres by age groups; (B) mean tIgA and IL-10 titres by [file pntd.0009455.s001.docx]

**Title: Associations of IL13 Gene Polymorphisms and immune factors with *Schistosoma haematobium* Infection in Schoolchildren in Four Schistosomiasis-endemic Communities in Ghana**

**Short title: IL13-gene polymorphism associations with *S. haematobium* infection in schoolchildren**

**SUPPORTING INFORMATION**

**
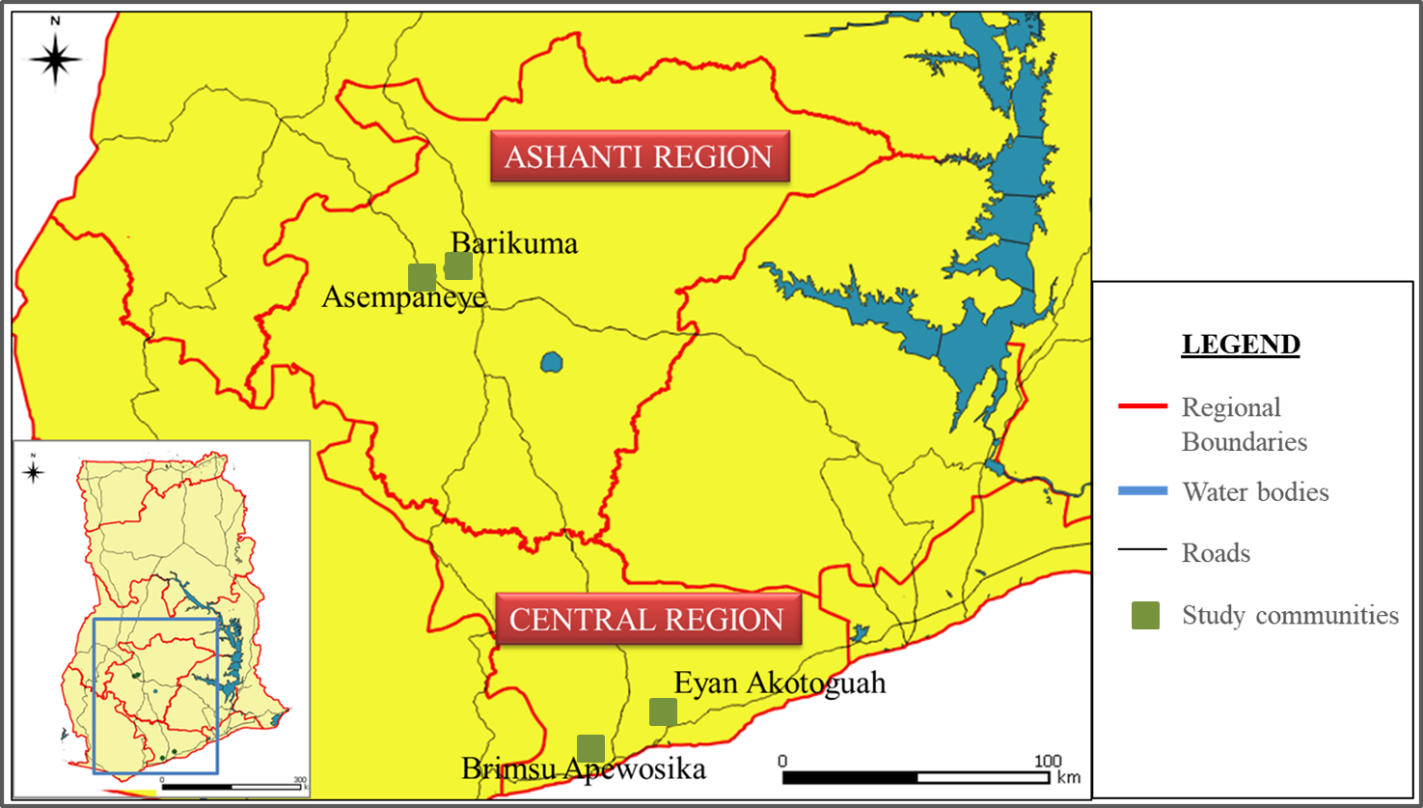
**

**Fig A:** A map of the Central and Ashanti Regions, indicating the locations of the study communities. Map was developed by co-author using the QGIS Girona version 3.0.3 (Boston, MA., USA). Shape files of the regions of Ghana were obtained online (URL: <https://github.com/tierney/gis-sandbox/tree/master/data/GIS-Ghana/ghana.shapefiles>). Also, GPS data were obtained from the field, using appropriate devices, and exported to Microsoft Office Excel version 2013, where conversions were made. The document was then exported to the QGIS Girona version 3.0.3 software as a delimited text file.

1. **IL13-1055**

Human species IL-13-1055 nucleotide sequence

**5’ATGCCTT GTGAGGAGGG TCAC** AAGCAC ACCCTTGTGA GGAGGTTGAG

CCCCATCGAG GACAGGACGG AGGGAGCCTG AGCAGGCAGA GAGGGGGCCT

GGGGAGGCGC TGGTTCGGGG AGGAAGTGGG TAGGGGAGAA ATCTTGACAT

CAACACCCAA CAGGCAAATG CCGTGGCCTC TGCTGTGGGG GTTTCTGGAG

GACTTCTAGG AAAACGAGGG AAGAGCAGGA AAAGGCGACA TGGCTGCAGG

GGCCAAGCCC AGGAGCCGCC CTCCACAGCA CTCATTCTGC AGAAGGGAAA

TTTGAGGCCC CCAGACGGCA GG**GGTTGATC CTGCAGAGAC TGG 3’**

Size of PCR products: 340 base pairs

Polymorphism expected: C to T

1. **IL13-591**

Human species IL-13-591 PCR nucleotide sequence

**5’ CCAGC CTGGCCCAGT TAAGAGTTTC** CCAGAAGGAT GGCCCATACA

CTTTAAATTA AAGGGGCCAG ACACGTGCAC ACTACTTCCA GCCACTCTGG

AAGCTGAGGT GGGGGGATCG CTTGAGTCTG GGAGTTGGAG GCCAGCCTAG

GCAGGCAACA TAGTGAGACC CCATCTCCAA AAAAACAAAA CAAAACAAAA

CAAAAAAACA CCAAAAAAGC TCCCAGAAAG ACCTCTGAAT CTTTCTGGAT

CTCTCAGTGG AGACCTGGAA ATCTGAACTT TGACAATCCC TCTCAC **AGTG**

**GGGCCA AGGA GGAATT AG 3’**

Size of PCR products: 313 base pairs

Polymorphism expected: A to G

1. **IL13-1258**

**5’ G GCCCTCTACT ACAGATTAGG AAACA**GGCCC GTAGAGGGGT CACACGGCCA

AGTAGCGGCA CTCCAGGCAC TGGGGGCCCT CGAGGGGAAG GGGCAGACTT

CTGGGAGTCA GAGCCAGCAG CTGGGCTGGG AAGCTTCGAG TGTGGACAGA

GAGGGTGGGA ATGACGTTCC CTGTGGGAAG AGAGGGTGGG CAAGCCTGGG

ATGCCTCTGA GCGGGAATCC AGCATGCCTT GTGAGGAGGG TCACAAGCAC

ACCCTTGTGA GGAGGTTG**AG CCCCATCGAG GACAGGACGG 3’**

Size of PCR products: 291 base pairs

Polymorphism expected: A to G

**Fig B:** Nucleotide sequences of the targeted sections of the IL-13 gene namely, (i) IL13-1055; (ii) IL13-591; and (iii) IL13-1258. Forward and reverse primer sequences for each of the targeted sections are in boldface. The target nucleotide for the single nucleotide polymorphism (SNP) in each section is underlined and red-lettered.


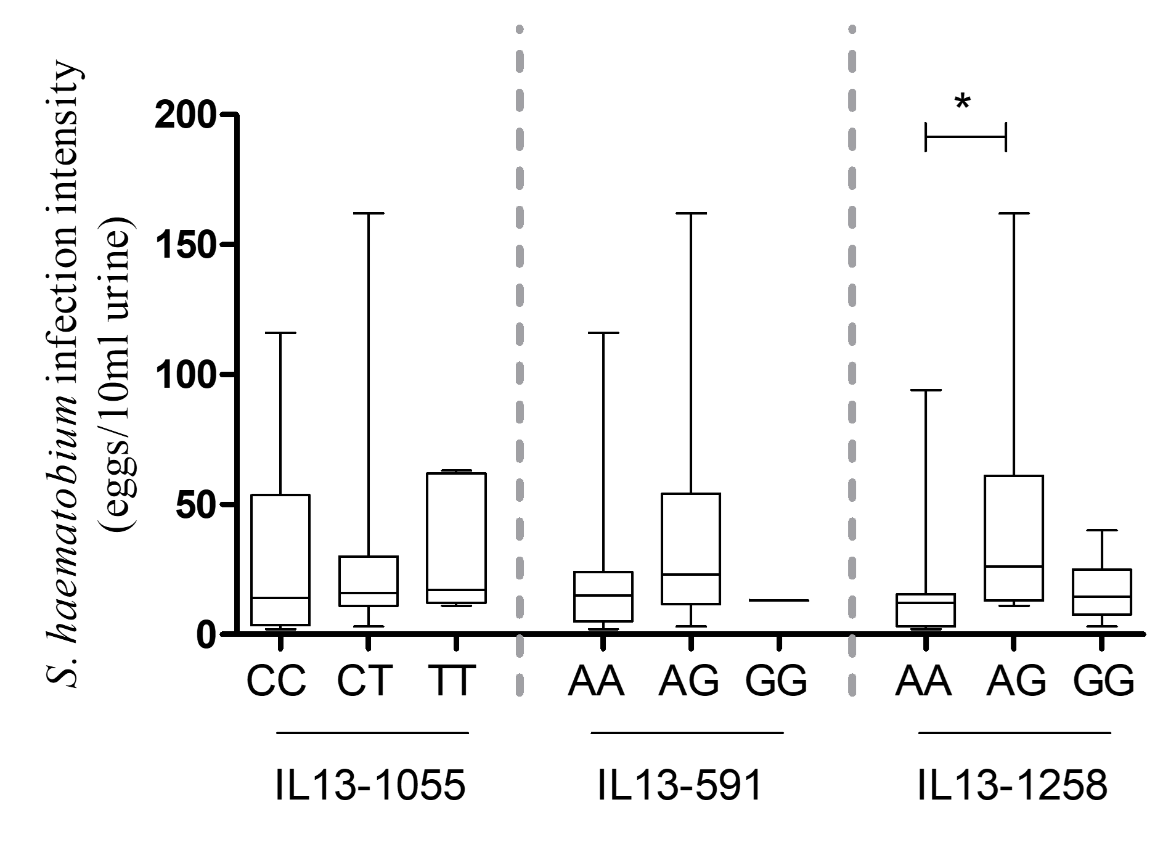


**Fig C:** Box and whisker plots depicting overall median *S. haematobium* infection intensity distribution for IL13-1055C/T, IL13-591A/G, and IL13-1258A/G polymorphisms. Comparisons between groups were done using the Kruskal-Wallis and Dunn’s post tests. Mid-horizontal line denotes median infection intensities. Upper and lower whiskers denote 95^th^ and 5^th^ percentiles respectively. ‘*’ denotes p-values < 0.05.


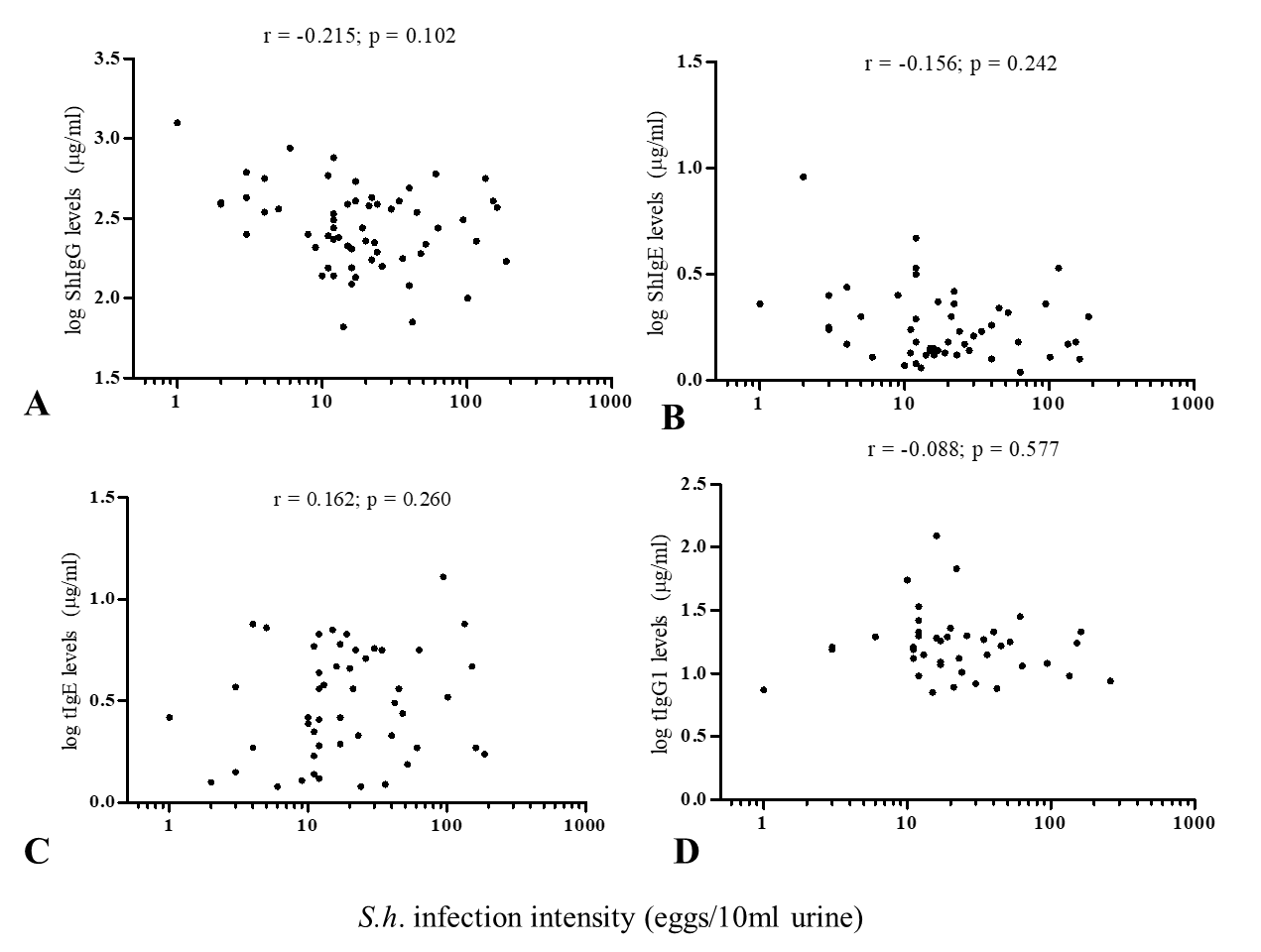

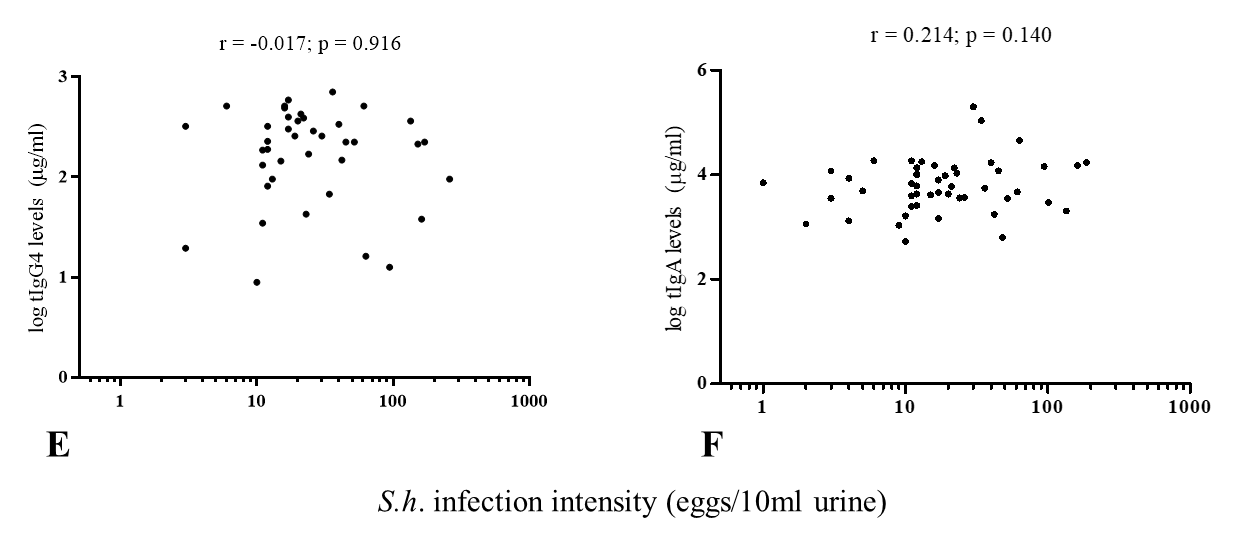


**Fig D:** Correlations of (A) ShIgG, (B) ShIgE, (C) tIgE, (D) tIgG1, (E) tIgG4, and (F) tIgA with S. haematobium infection intensity. ‘r’ denotes the Spearman’s rank correlation coefficient (Spearman’s rho).


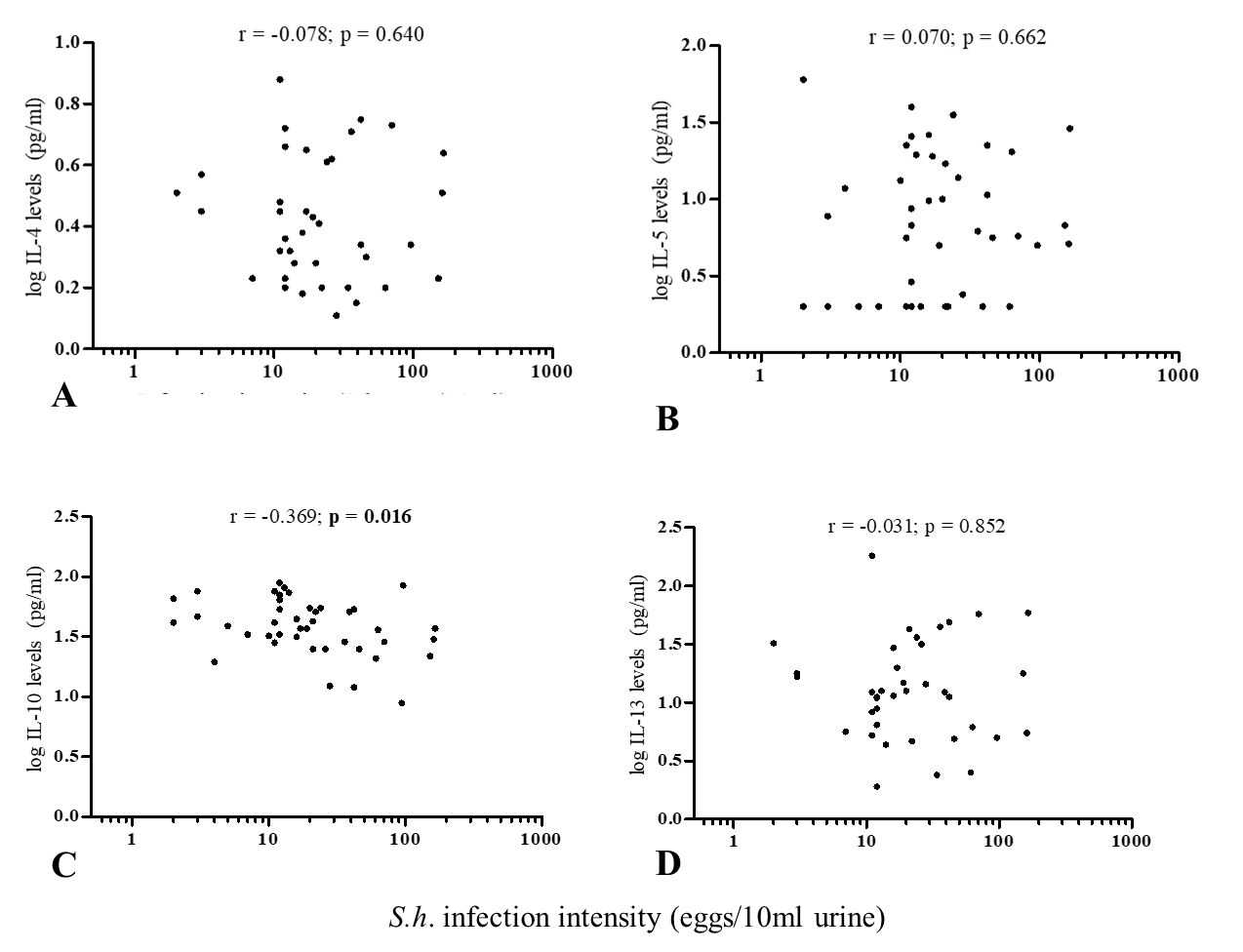


**Fig E:** Correlations of (A) IL-4, (B) IL-5, (C) IL-10, and (D) IL-13 with *S. haematobium* infection intensity. ‘r’ denotes the Spearman’s rank correlation coefficient (Spearman’s rho). Significant correlations are in boldface.


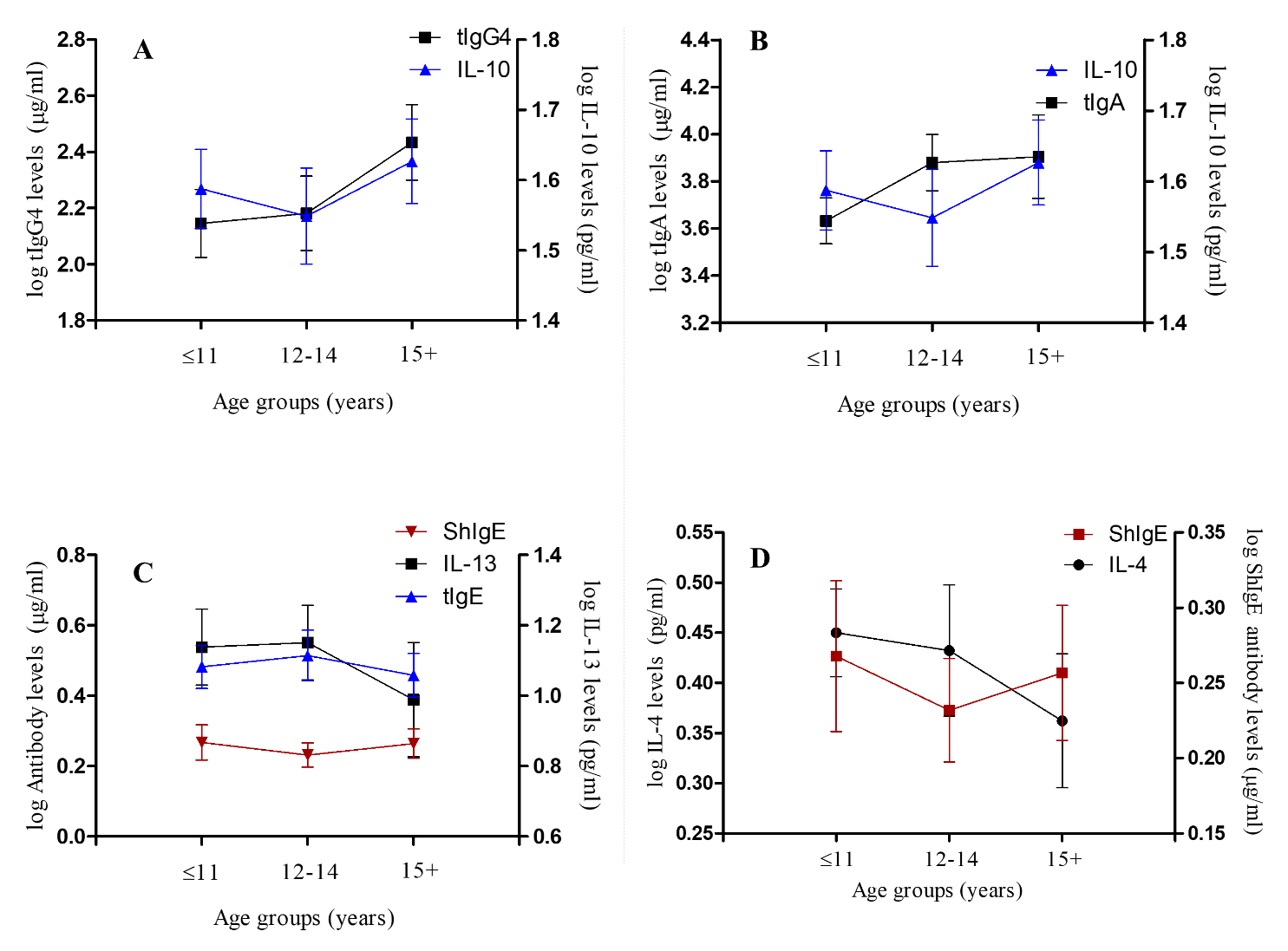


**Fig F:** Stratification of measured plasma levels of (A & B) anti-inflammatory immune factors; and (C &D) pro-inflammatory immune factors by age groups. (A) Mean IL-10 and tIgG4 titres by age groups; (B) mean tIgA and IL-10 titres by age groups; (C) mean IL-13, tIgE, and ShIgE titres by age groups; and (D) mean IL-4 and ShIgE titres by age groups. Error bars represent the standard error of mean (SEM).
